# Seed Detection and Discrimination by Ground Beetles (Coleoptera: Carabidae) Are Associated with Olfactory Cues

**DOI:** 10.1371/journal.pone.0170593

**Published:** 2017-01-20

**Authors:** Sharavari S. Kulkarni, Lloyd M. Dosdall, John R. Spence, Christian J. Willenborg

**Affiliations:** 1 Department of Agricultural, Food, and Nutritional Science, University of Alberta, Edmonton, Alberta, Canada; 2 Department of Renewable Resources, University of Alberta, Edmonton, Alberta, Canada; 3 Department of Plant Sciences, College of Agriculture and Bioresources, University of Saskatchewan, Saskatoon, Saskatchewan, Canada; Rutgers The State University of New Jersey, UNITED STATES

## Abstract

Olfactory ability is an element of fitness in many animals, guiding choices among alternatives such as mating partners or food. Ground beetles (Coleoptera; Carabidae), exhibit preferences for prey, and some species are well-known weed seed predators. We used olfactometer-based bioassays to determine if olfactory stimuli are associated with detection of *Brassica napus* L., *Sinapis arvensis* L., and *Thlaspi arvense* L. seeds by ground beetles characteristic of agroecosystems, and whether behavioural responses to seed odors depended on seed physiological state (imbibed or unimbibed). Imbibed *B*.*napus* seeds were preferred over other weed species by two of the three carabid species tested. Only *A*. *littoralis* responded significantly to unimbibed seeds of *B*. *napus*. Sensitivity to olfactory cues appeared to be highly specific as all carabid species discriminated between the olfactory cues of imbibed brassicaceous weed seeds, but did not discriminate between weed seeds that were unimbibed. Overall, our data suggest that depending on seed physiological state, odours can play an important role in the ability of carabids to find and recognize seeds of particular weed species.

## Introduction

Carabid beetles (Coleoptera: Carabidae) are invertebrate predators, some of which consume weed seeds in temperate agroecosystems [1–3]. In fact, post-dispersal seed consumption by carabids can limit population growth of weedy plants [4, 5] and affects the population dynamics of weeds in agroecosystems [5,6]. However, the extent to which seed predation constrains weed population growth depends on the ability of predators to detect them [6–13], and little is known about this process.

Most invertebrate predators use a variety of sensory cues in localizing prey or habitats in which prey may be found [14–16]. Ground beetles assess habitat suitability using a range of cues and, it is well known that volatile chemicals associated with the habitat can be involved [17,18]. Granivorous carabids also use tactile stimuli associated with seeds, particularly seed structural strength and physical density in seed detection [19]. Volatile compounds associated with hosts or prey items provide olfactory cues that help many invertebrates orient to food [20, 21], and perception of such olfactory cues also play an important role in detecting food patches or selecting sites for oviposition among ground beetles [14, 21, 22].

Despite the probable importance of olfactory cues in organizing these behaviours, research about the possible role of olfaction in seed detection has been limited. For example, it remains uncertain whether visual cues, olfactory cues, or a combination of both guides seed detection and selection. A recent study [23] suggests that volatile compounds emitted by imbibed seeds influence weed seed detection in the ground beetle, *Harapalus pensylvanicus* DeGeer; however, potential differences among carabid species in response to olfactory cue recognition and in seed preferences have not been well explored.

Many dormant weed seeds persist in the soil seedbank either partially or completely imbibed [23]. *B*. *napus* seeds, for example, can remain dormant in an imbibed state for up to five years [24]. As a result, weed seed predators such as carabids are likely to be exposed to both dry and imbibed seeds during foraging. Whether seeds are imbibed is an important consideration for seed predation research because imbibition triggers chemical processes in the seed that can result in the release of volatile compounds such as ethanol and acetaldehyde [25, 26, 27]. Release of these cues may, in turn, affect seed discovery and ultimately, seed consumption. Elucidating such cues can help us better understand mechanisms of seed detection, and thus, the probability of species consuming various weed seeds in different environments.

Carabids are able to detect weed seeds that are buried in soil [13, 28, 29], suggesting that adult beetles use cues in addition to visual or tactile stimuli to detect seeds. However, both the mechanisms underlying weed seed detection and the potential role of olfactory cues are unknown for a significant proportion of carabid species that consume weed seeds. In order to better understand these relationships, we investigated whether three omnivorous carabid species used olfactory cues to detect the presence of seeds of brassicaceous weed species, and whether beetle responses to olfactory cues differed between unimbibed and imbibed seeds. We tested beetle preferences for both imbibed and unimbibed seeds in olfactory bioassays, testing the hypothesis that cues associated with seed odors lead to behavioural responses in adult carabids that depend on seed physiological state.

## Materials and Methods

### Carabid species

The carabid beetles used in the study were collected in weedy patches and canola (*Brassica napus* L.) field margins using dry pitfall traps (12 cm diameter by 14 cm depth). All collections were made during the summers of 2012 and 2013 at the South Campus (53.50° N, 113.52° W) and Ellerslie (53.25° N, 113.33° W) Research Stations of the University of Alberta, Edmonton, Canada. For experiments described here, we used adults of three omnivorous carabid species, *Amara littoralis* Mannerheim, *Harpalus affinis* Schrank, and *Pterostichus melanarius* Illiger, all of which are common in agroecosystems of central Alberta, and known to feed on weed seeds. After capture, adult beetles were held in plastic containers (Gladware^®^, 14 cm x 12 cm x 10 cm, 1.89 L capacity) at 21°C with a 16 h photoperiod in the laboratory, and were starved for 48 h, prior to use in the bioassay experiments described below.

### Olfactory bioassays

To investigate whether olfactory cues associated with particular seeds played a role in seed foraging, we used both multi-choice and two-choice bioassays. Seeds of three brassicaceous weed species [*Brassica napus* L. (considered weedy volunteers), wild mustard, *Sinapis arvensis* L., and field pennycress, *Thlaspi arvense* L.] were collected from the sites mentioned above and stored at 5°C for seven months prior to use in the bioassays. All three weed species are common in local agroecosystems [30] and their seeds are readily consumed by carabids [31].

**Fig 1:**
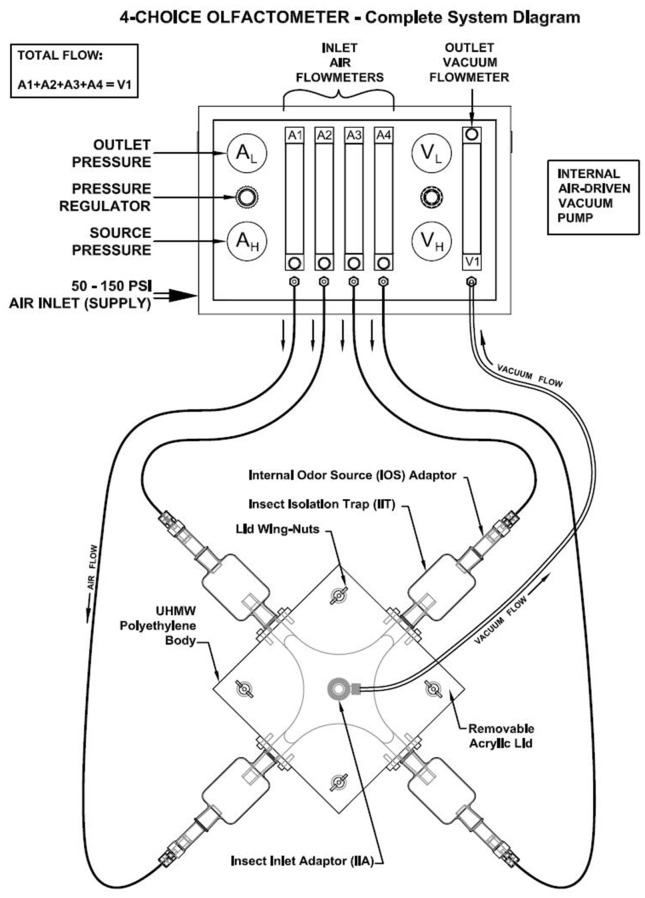
Schematic of a four chambered olfactometer. The main choice arena of the olfactometer measured 30.48 x 30.48 x 2.54 cm, and was covered with a removable lid. It consisted of four outlet ports laterally connected to four odour source chambers, and a ventral insect inlet port to introduce the test insect. Each lateral outlet port was connected to the internal odour source (IOS) with a glass insect isolation trap (IIT). The odour source was connected to an air delivery system that pumped moist air through the odour sources to the choice arena, and a vacuum to the insect inlet chamber to centralize the airflow throughout the choice arena. The rate of air delivery was 1 L/min. The air from all the odour sources was directed to the insect inlet chamber using a vacuum suction mechanism, and this exposed the test insect to odours emanating from different chambers, allowing it to make a choice. Image source: ARS, Gainesville, Florida.

Laboratory bioassays were conducted using a four-chambered olfactometer (Analytical Research Systems, Gainesville, Florida, USA, Model #OLFM-4C-2440PE, Fig 1). Single carabids were introduced into the insect inlet adapter (IIA) of the olfactometer (Fig 1) and given 20 minutes to orient and choose a ‘preferred’ odour source chamber from among four treatments. We conducted two sets of experiments as described below.

In the first set of bioassays, the treatments were three 20 mg masses of seeds of *B*. *napus*, *S*. *arvensis* and *T*. *arvense*, respectively, placed on a piece of filter paper in one of the chambers, and a similar blank filter paper without seeds provided as a control. All trials were replicated 40 times, using either imbibed or unimbibed seeds of all three weed species, each time with naïve beetles.

We assumed that beetles either chose insect isolation traps (IITs) at random or that their choices reflected odours emanating from different seeds held in the internal odour source (IOS). We observed the behavior of each beetle and recorded the times when it entered any IIT and exited this chamber and into the main arena. Absence of specific orientation behavior or apparently random walking movements, e.g., entry and immediate retreat from various IITs, or a lack of any movement in the olfactometer arena, were scored as lack of choice. For each insect tested, data were recorded for choice of IIT and ‘residence time’ (time in minutes spent in each IIT). The longest residence time was taken to be the ‘choice’ of each individual beetle.

Treatments were assigned randomly to the odour chambers for replicate trials, and positions of the chambers were changed every five runs to minimize bias resulting from orientation of the apparatus. Internal odour sources (IOS) and insect isolation traps IITs were rinsed with 70% ethanol and dried between runs to minimize effects of any residual odours. Olfactory bioassays were conducted under red light (Philips 23 W PAR38 Red light) to simulate nocturnal conditions in the field [32].

Given the overall preference observed among all three carabid species for seeds of *B*. *napus* [31], we employed a second bioassay to further understand how preferences were affected by imbibition by directly comparing responses between imbibed and unimbibed seeds in a two-choice olfactometer. For these experiments, we converted the olfactometer to function as a two-chambered arena by plugging and stopping airflow to two of the odour inlets. In one of the two operational chambers, we placed 200 mg of unimbibed *B*. *napus* seed, while in the other we placed the same mass of imbibed seeds. As above, 40 individual beetles of each species were tested.

### Statistical analyses

A chi-square (PROC FREQ, SAS 9.3) test was used to analyze data from the first (four-chambered) olfactometer experiments. To identify if preferences existed among the odours emanating from imbibed and unimbibed seeds of the three weed species, residence time for each weed seed species and a control (four-choice assay) were compared with mixed-model ANOVA (PROC MIXED, SAS 9.3) [33], using residence time as the response variable to be predicted by weed species. Analyses were carried out separately for both imbibed and unimbibed seeds for each carabid species, with weed species treated as a fixed effect.

Data about imbibed seeds did not meet the assumptions of ANOVA; therefore, we fitted generalized estimating equations (GEEs) to these data using PROC GLIMMIX (SAS 9.3) with a negative binomial error distribution function (PROC GLIMMIX) [33]. Numerous possible error distribution functions were tested, including the Poisson distribution, but the negative binomial error function provided the best fit (based on the ratio of Chi-square/df ratio being close to 1). We tested models similar to those for unimbibed seeds.

Differences in mean residence times among weed species were compared using Tukey’s *post-hoc* test. Data from the second bioassay comparing responses to unimbibed and imbibed *B*. *napus* seeds were analyzed using a chi-square test (PROC FREQ) to establish if preferences existed.

## Results

**Fig 2:**
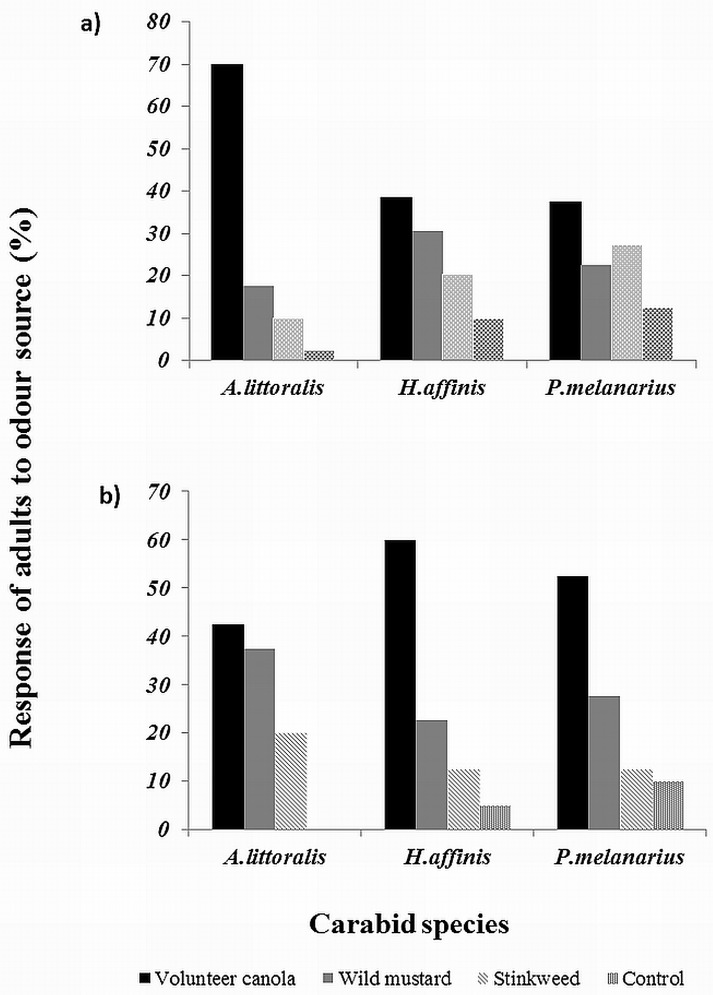
Response of the adults (%) of *P*. *melanarius*, *A*. *littoralis* and *H*. *affinis* responding to odour emanating from a) unimbibed seeds and b) imbibed seeds of *B*.*napus*, *S*.*arvensis*, *T*. *arvense* and control chambers of a four chambered olfactometer.

More adults of all three carabid species responded numerically to odours from unimbibed seeds of *B*. *napus* in the four-choice bioassays than to other weed species (Fig 2a), and this was especially pronounced statistically for *A*. *littoralis* adults (χ^2^ = 45, *P* < 0.0001). Although not statistically significant, a similar trend was observed for *H*. *affinis* (χ^2^ = 7.05, *P* = 0.07), while no statistically significant differences in response to odors were observed for *P*. *melanarius* (χ^2^ = 5.2, *P* = 0.16).

**Fig 3:**
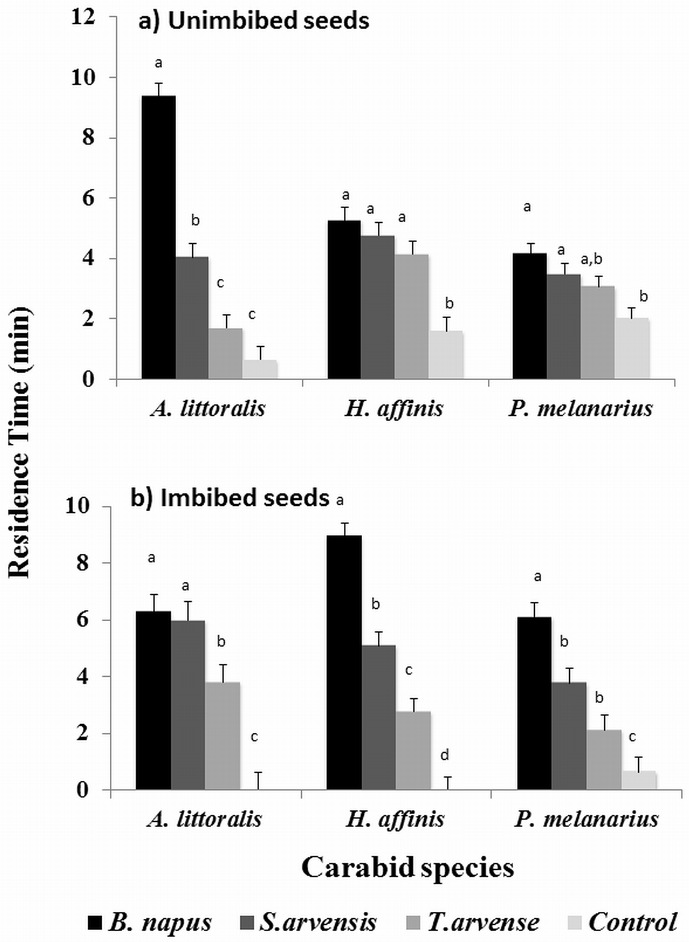
Mean residence time i.e. time spent by the carabid species *P*. *melanarius*, *A*. *littoralis* and *H*. *affinis* in the odour chamber from a) unimbibed seeds and b) imbibed seeds of *B*.*napus*, *S*.*arvensis*, *T*. *arvense* and control chambers of a four chambered olfactometer. For a given carabid species, treatment means with different letters indicate significant differences in percent response to odours from different seed treatments using Tukey’s test.

Residence times were significantly longer in response to unimbibed seed odour than to controls for *P*. *melanarius* (*F* = 4.51; df = 3, 117; *P* = 0.004), *H*. *affinis* (*F* = 13.53, df = 3, 114; *P* = 0.001 Fig 3a;) and *A*. *littoralis* (*F* = 86.12; df = 3, 117; *P* = 0.0001). Post-hoc tests reveal, however, that residence times differed significantly among the three weed species for only *A*. *littoralis*. Adults of *A*. *littoralis* spent approximately 10, 4, and 1 minutes in the chambers with odors of unimbibed seeds of *B*. *napus*, *S*. *arvensis* and *T*. *arvense*, respectively. Residence times in chambers with *B*. *napus* were significantly greater than in chambers with *S*. *arvensis*, *T*. *arvense* or controls. Interestingly, responses of *A*. *littoralis* did not differ between the control and *T*. *arvense*, but both treatments prompted significantly less response than did odours from *B*. *napus* and *S*. *arvensis*.

Responses to odours from imbibed seeds of the three weed species also varied among carabid species, but in a different way (Fig 2b). A greater percentage of both *P*. *melanarius* (χ^2^ = 15.6, *P* < 0.001) and *H*. *affinis* (χ^2^ = 17.1, *P* < 0.001) responded to odours of *B*. *napus* compared with *S*. *arvensis* and *T*. *arvense*, and both species responded more strongly to *S*. *arvensis* than to *T*. *arvense* (Fig 2b). For example, 52% of *P*. *melanarius* adults responded to odours from *B*. *napus* compared to 25% from *S*. *arvensis* and only 16% from *T*. *arvense*. Interestingly, the percentage of *A*. *littoralis* adults responding to different odour sources from imbibed seeds did not differ significantly among weed species (χ^2^ = 2.1; *P* = 0.34), although the same trend was observed among the mean responses (i.e., *B*. *napus* > *S*. *arvensis* > *T*. *arvense*) (Fig 2b).

Overall response, measured as residence times, differed among odour treatments for imbibed seeds in all three species: *P*. *melanarius* (*F* = 18.11, df = 3, 108; *P* <0.0001), *A*. *littoralis* (*F* = 19.53, df = 3, 117; *P* < 0.0001) and *H*. *affinis* (*F* = 34.02, df = 3,117; *P* < 0.0001) (Fig 3b). Although mean residence times did not differ among weed species for *A*. *littoralis*, responses to all weed species differed from those to the control (0 minutes). Both *P*. *melanarius* and *H*. *affinis* spent longer times in the odour chamber of *B*. *napus* seeds than the remaining two weed species and control.

**Fig 4:**
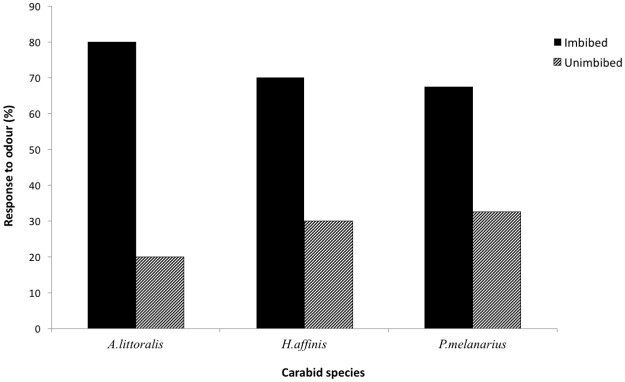
Response of the adults (%) of *P*. *melanarius*, *A*. *littoralis* and *H*. *affinis* responding to odour emanating from unimbibed and imbibed seeds of *B*.*napus* in a two choice bioassay.

To explicitly compare carabid responses to unimbibed and imbibed seeds, we compared these in two-choice bioassays using seeds of *B*. *napus*. In these two-choice experiments adults of all three species showed a stronger response to imbibed seeds (*P*. *melanarius*: χ^2^ = 8.10, *P* < 0.01; *A*. *littoralis*: χ^2^ = 14.40, *P* < 0.0001; *H*. *affinis*: χ^2^ = 6.40, *P* = 0.01) compared to unimbibed seeds (Fig 4).

## Discussion

Carabid beetles of three species common in prairie agroecosystems of western Canada exhibited different behavioral responses to olfactory cues associated with the seeds of weed species also common in those ecosystems. In bioassays of response to odours of both unimbibed and imbibed seeds, *B*. *napus* seeds were most preferred overall, followed by those of *S*. *arvensis* and *T*. *arvense*, respectively. Because these carabids also exhibited the same order of preference for consumption of these species in previous experiments [31], we reason that olfactory cues are being used in seed detection. Taken together, and assuming that response to odours should be strongest for preferred seeds, these results show that carabid species differ in their physiological abilities to detect particular odours associated strictly with seeds alone. This may not necessarily be associated with differences in ability to find seeds under field conditions, however, as ecological setting may also influence the ability of beetles to detect weed seeds [23].

Response of all carabids to olfactory cues was much enhanced by seed imbibition. In fact, two of the three species included in our study, *P*. *melanarius* and *H*. *affinis*, responded significantly only to odours emanating from imbibed seeds. Although *A*. *littoralis* responded to odours emanating from both dry and imbibed seeds, the beetles of this species responded more strongly to odours associated with imbibed seeds of *B*. *napus* compared with unimbibed seeds. Thus, preferences for imbibed seeds of various weed species appear to vary among carabid species.

Residence time in an odour chamber has been frequently used to indicate the attractiveness of an odour to the test species [14], and longer residence times have been interpreted as arrestment caused by an odour [20]. In our study, *A*. *littoralis* had longer residence times in chambers with odours emanating from either unimbibed or imbibed *B*. *napus* seeds, while both *P*. *melanarius* and *H*. *affinis* showed greater arrestment only in response to imbibed *B*. *napus* seeds. This suggests that arrestment responses of carabids may be a function of both the physiological state of the seed and the carabid species tested.

Although we know that olfactory cues can be significant in carabid detection of invertebrate prey [21, 34, 35], relatively little is known about behavioural responses of carabids to potential olfactory cues emitted by weed seeds. Better understanding differential responses of beetles to seed odours has implications for understanding weed seed predation under field conditions. For example, the extent to which carabids will affect the fate of weed seeds, particularly unimbibed, dormant seeds in the seedbank, will be determined by the capacity of individual species to detect weed patches. The fate of imbibed seeds, on the other hand, is more likely to be a function of weed species as opposed to composition of carabid species assemblages. Weed species such as *B*. *napus*, for example, appear more likely than other brassicaceous weeds to be detected by seed predators, such as *A*. *littoralis* and *H*. *affinis*, that are prominent in canola fields.

Our research indicates that the carabid species tested respond differentially to weed seed odors, in the absence of other cues. In addition, we have shown that carabid responses to seeds are affected by the physiological state of seeds. In particular, seed imbibition increased the attraction to olfactory signals emanating from seeds of all weed species. Variation in the sensitivity of carabids to olfactory cues from three common brassicaceous weed species could be associated with discrimination among weed species in the field by carabid seed predators.

## Supporting Information

S1 File Raw data used for chi-square tests on dry and imbibed seed (Fig 2). (XLSX)

S2 File Raw data used for analyses of residence time for dry seeds (Fig 3a). (XLSX)

S3 File Raw data used for analyses of residence time for imbibed seeds (Fig 3b). (XLSX)

S4 File Raw data used for two-choice bioassay chi-square tests (Fig 4). (XLSX)
